# Identification of an FHL1 Protein Complex Containing Gamma-Actin and Non-Muscle Myosin IIB by Analysis of Protein-Protein Interactions

**DOI:** 10.1371/journal.pone.0079551

**Published:** 2013-11-12

**Authors:** Lili Wang, Jianing Miao, Lianyong Li, Di Wu, Yi Zhang, Zhaohong Peng, Lijun Zhang, Zhengwei Yuan, Kailai Sun

**Affiliations:** 1 Key Laboratory of Health Ministry for Congenital Malformation, Shengjing Hospital, China Medical University, Shenyang, China; 2 Department of Pediatric Surgery, Shengjing Hospital, China Medical University, Shenyang, China; 3 Department of Medical Genetics, China Medical University, Shenyang, China; New York Medical College, United States of America

## Abstract

FHL1 is multifunctional and serves as a modular protein binding interface to mediate protein-protein interactions. In skeletal muscle, FHL1 is involved in sarcomere assembly, differentiation, growth, and biomechanical stress. Muscle abnormalities may play a major role in congenital clubfoot (CCF) deformity during fetal development. Thus, identifying the interactions of FHL1 could provide important new insights into its functional role in both skeletal muscle development and CCF pathogenesis. Using proteins derived from rat L6GNR4 myoblastocytes, we detected FHL1 interacting proteins by immunoprecipitation. Samples were analyzed by liquid chromatography mass spectrometry (LC-MS). Dynamic gene expression of FHL1 was studied. Additionally, the expression of the possible interacting proteins gamma-actin and non-muscle myosin IIB, which were isolated from the lower limbs of E14, E15, E17, E18, E20 rat embryos or from adult skeletal muscle was analyzed. Potential interacting proteins isolated from E17 lower limbs were verified by immunoprecipitation, and co-localization in adult gastrocnemius muscle was visualized by fluorescence microscopy. FHL1 expression was associated with skeletal muscle differentiation. E17 was found to be the critical time-point for skeletal muscle differentiation in the lower limbs of rat embryos. We also identified gamma-actin and non-muscle myosin IIB as potential binding partners of FHL1, and both were expressed in adult skeletal muscle. We then demonstrated that FHL1 exists as part of a complex, which binds gamma-actin and non-muscle myosin IIB.

## Introduction

The *Fhl1* gene is located on chromosome Xq36, and encodes four-and-a-half LIM protein-1 (FHL1) and its spliced isoforms, SLIMMER and FHL1C [1]. FHL1 is a multifunctional protein, characterized by the tandem arrangement of four-and-a-half highly conserved LIM domains. Northern blot analysis has confirmed strikingly high expression of FHL1 in skeletal muscle and heart, and markedly lower expression levels in several other tissues, including the colon, small intestine, and prostate [2], [3].

LIM domains are capable of interacting with other LIM domain proteins, where they form homo- or heterodimers. LIM domains also associate with tyrosine-containing motifs, PDZ domains, ankyrin repeats, and helix-loop-helix domains [4]. Previous studies have verified that FHL1 and its interacting proteins are associated with several signaling pathways, including those that are integrin-mediated, mitogen-activated protein kinase-mediated, beta-adrenergic receptor transduced, G-protein coupled receptor transduced, pathways mediated by NFATc1, transforming growth factor-β like signaling pathways, and estrogen receptor signaling pathways [5]–[10]. It has been indicated that the interaction between ACTN1 and FHL1 is a critical coupling event in the regulation of actin-based stress fiber structures [11]. Skeletal muscle tissue contains slow as well as fast twitch muscle fibers, which possess different metabolic and contractile properties. FHL1 is located at the Z-disc in skeletal muscle, and is involved in sarcomere assembly, muscle differentiation, growth, and biomechanical stress responses [12]–[15]. Mice lacking *Fhl1* were protected from the onset of hypertrophic cardiomyopathy, which is normally induced by biomechanical stress, whereas transgenic expression of *Fhl1* in mice promoted skeletal muscle hypertrophy [13], [16]–[20]. Twenty-seven mutations have been identified in the *FHL1* gene that contribute to the development of six different myopathies, each of which present a combination of various protein aggregates, joint contractures, muscle atrophy/hypertrophy, and cardiovascular diseases [4]. These observations suggest that *FHL1* plays an important role in muscle growth and development.

Idiopathic congenital clubfoot (CCF, MIM119800) is a congenital limb deformity, which is characterized by skeletal muscle abnormalities [21], [22]. Muscle abnormalities classified as congenital fiber type disproportion (slow fiber increase and fast fiber decrease), or additional muscle bundle in the gastrocnemius, have been found in many CCF cases, which may predict recurrent limb deformities [23]–[29]. Our previous work showed that expression of FHL1 was downregulated in musculus flexor hallucis longus of congenital clubfoot, which demonstrated that downregulation in FHL1 expression is involved in the formation of skeletal muscle abnormalities in CCF [21]. However, the molecular mechanisms whereby FHL1 contributes to skeletal muscle differentiation, myotube formation during embryo development and the pathology of CCF remains unknown. Since the functional properties of FHL1 are likely to be mediated by a diversity of interacting partners, the study of FHL1 protein interactions in skeletal muscle development may provide new insights into its functional role in CCF pathogenesis and other FHL1-induced myopathies. Here, we show that FHL1 exists as an integral component of a complex that includes gamma-actin (*Actg1*) and non-muscle myosin IIB (*Myh10*).

## Materials and Methods

### IRB: NO.2013PS06K

All animals in this study were from the Animal Center of Shengjing Hospital at China Medical University. Pregnant female rats or adult wild-type rats were anesthetized and killed by cervical dislocation. All studies were performed in accordance with the protocol approved by the Institutional Animal Care and Use Committee of the China Medical University for Basic Research in Developmental Disabilities. All surgery was performed under anesthesia, and all efforts were made to minimize suffering.

### Reagents

Cell culture reagents were obtained from Gibco (Shanghai,America). Mouse anti-FHL1 (WH0002273M1), was used in Western immunoblots, mouse anti-skeletal myosin (Fast, M4276) and mouse anti-skeletal myosin (Slow, M8421) antibodies were obtained from Sigma-Aldrich (Shanghai,America). Goat anti-FHL1 (sc-23176) was used in immunofluorescence co-staining. Mouse anti-myh10 (sc-376942) and mouse anti-gamma-actin (sc-65637) antibodies were obtained from Santa Cruz (Santa Cruz,California,USA). Additional antibodies included Texas Red-conjugated rabbit anti-goat and FITC-conjugated donkey anti-mouse secondary antibodies (Protein Tech) that were used for immunolocalization studies.

### L6GNR4 cell culture

Rat myoblastocytes (L6GNR4, obtained from the Cell Laboratory, Chinese Academy of Sciences, Shanghai) were cultured in Dulbecco’s modified Eagle’s medium supplemented with 10% fetal calf serum (FCS), 100 units/mL penicillin, and 100 µg/mL streptomycin.

### Immunoprecipitation

Total myoblastocyte (L6GNR4) protein (500 mg) was pre-cleared for 1 h at 4°C with 20 µL of protein A/G beads (Sigma), with the beads pelleted by centrifugation. FHL1 antibody (2.5 mg) or mouse IgG (2.5 mg, as the negative control) or mouse GAPDH antibody (2.5 mg, as the negative control) was incubated with 30 µL of protein A/G beads and 500 µL 1× PBS for 1 h at 4°C. Antibody/pre-immune sera-bound beads were pelleted, washed four times with solubilization buffer (50 mM Tris–HCl, pH 7.4, 100 mM NaCl, 0.32 M sucrose, 5 mM EDTA, 2.5 mM EGTA, 1% Triton X-100, and protease inhibitors (1 mM PMSF, 1 mM AEBSF, 10 mg/mL leupeptin, 10 mg/mL pepstatin), following which the beads were resuspended in 100 µL of solubilization buffer. The pre-cleared cytosolic fraction and protease inhibitors were added to the antibody-bound beads in solubilization buffer and incubated with rocking at 4°C overnight. The beads were then pelleted, washed four times in RIPA buffer (20 mM Tris–HCl, pH 7.4, 137 mM NaCl, 10% glucose, 0.1% SDS, 0.5% Na-deoxycholate, 1% Triton X-100, 2 mM EDTA, 1 mM PMSF, 20 mM leupeptin), followed by four washes in solubilization buffer. Sample buffer (5 µL, 4×) was added to all specimens, and samples were heated to 95°C for 5 min. Proteins were separated by sodium dodecyl sulfate-polyacrylamide gel electrophoresis (SDS-PAGE, 12% gel).

### Mass spectrometry and protein identification

CBB-stained gels were scanned by a PowerLook 2100XL image scanner (Umax, Taiwan). FHL1 antibody immunoreactive bands were selected and excised manually from the gel for further analysis. CBB-stained bands were destained in 50% acetonitrile (ACN)/25 mM ammonium bicarbonate buffer and dried by SpeedVac. The dried gel fragments were completely immersed and re-hydrated in trypsin solution (15 µg/mL) for 1 h at 4°C, followed by the addition of 5 mL of 25 mM ammonium bicarbonate buffer. After incubation for 16 h at 37°C, the peptides were digested and extracted from the gel fragments by a separate incubation in 5% trifluoroacetic acid (TFA) and 2.5% TFA/50% ACN at 37°C for 1 h. The trypsin digested peptides were finally dissolved in MALDI matrix (5 mg/m α-cyana-4-hydroxycinnamic acid in 0.1% TFA and 50% ACN), spotted onto 192-well stainless steel MALDI target plates, and analyzed using an ABI 4800 Proteomics Analyzer MALDI-TOF/TOF mass spectrometer (Applied Biosystems, USA). The MS and MS/MS spectra were searched against the International Protein Index (IPI) rat database (version 3.18) using the GPS Explorer TM v3.0 and MASCOT database search algorithms (version 2.0). The search criteria used in this analysis were: trypsin specificity, cysteine carbamidomethylation (C) and methionine oxidation (M) as variable modifications; one trypsin miscleavage allowed; 0.2-Da MS tolerance; and 0.3-Da MS/MS tolerance. Positive identification of proteins was accepted with a MOWSE score ≥ 58 and a statistical significance of *P* < 0.05.

### FHL1, gamma-actin and non-muscle myosin IIB expression in rat embryos

Lower limb protein extracts were prepared from E14, E15, E17, E18, and E20 rat embryos. The protein concentration of each lysate was determined by the bicinchoninic acid (BCA) assay according to the manufacturer's guidelines. Total proteins (90 µg) were separated by SDS-PAGE (12% gel) and transferred to polyvinylidene fluoride (PVDF) membranes. Membranes were washed in Tris-buffered saline (TBS) containing 0.1% Tween-20, and then incubated with specific primary antibodies (anti-FHL1, WH0002273M1, 1∶2000; anti-fast skeletal myosin, M4276, 1∶2000; anti-slow skeletal myosin, M8421, 1∶2000; anti-myh10, sc-376942, 1:500; anti-gamma-actin, sc-65637, 1∶1000) followed by incubation with secondary antibody (diluted 1:2000). GAPDH protein was used as an internal positive control.

### *Fhl1*, *Actg1*, and *Myh10* RNA expression in adult skeletal muscle

*Myh10* (NMHC II-B) expression in adult skeletal muscle is controversial [30], [31]. Thus, we analyzed and confirmed the expression of *Fhl1*, *Actg1*, and *Myh10* in adult skeletal muscle. In addition, RNA extracted from lower limbs of E17rat embryos or from L6GNR4 cells was used as positive control. RNA was extracted by the TRIzol Reagent procedure according to the manufacturer’s protocol. cDNA synthesis was initiated with 3 µg of RNA using the TaKaRa One Step RNA PCR kit (AMV) (TaKaRa Biotechnology, Dalian, JAPAN). Actin was used as an endogenous “housekeeping gene” control. Primers used in the RT-PCR procedure are shown in Table 1.

**Table 1:** Primers used in RT-PCR.

Gene	Primer Sequence	Product length	Annealing Tm
*Actin*	GGAGATTACTGCCCTGGCTCCTA	139 bp	56°C
	GACTCATCGTACTCCTGCTTGCTG		
*Fhl1*	CGTGCCAGTAGCGATTCTTAT	107 bp	56°C
	GCTGCCTGAAGTGCTTTGAC		
*Actg1*	AATGCCGTGCTCAATAGGGT	229 bp	56°C
	CGCAATGGAAGAAGAAATCG		
*Myh10*	ACTTGCCAAAGCGAGATGAG	129 bp	54°C
	ACAAAGGAAGAAAGGACCAC		

### FHL1 expression in adult skeletal muscle fibers

Wild-type adult rat gastrocnemius muscle tissues were dissected from the center of the lateral head of the muscle. All resected specimens were fixed in 10% neutral buffered formalin (pH 7.4), embedded in paraffin, and cut into 5 µm sections. For immunofluorescence analysis, non-specific interactions were first blocked in 10% FBS and permeabilization buffer (0.2% Tween-20, 0.5% Triton X-100 in PBS pH 7.0) for 30 min. Goat anti-rat FHL1 antibody (sc-23176) was used in this and subsequent immunofluorescence procedures for simultaneous detection of two proteins. The sections were incubated in primary antibodies (anti-FHL1, sc-23176, 1:100; anti-fast skeletal myosin, M4276, 1:200; anti-slow skeletal myosin, M8421, 1:200) that were diluted in permeabilization buffer, and incubated overnight at 4°C. Sections were then washed three times in PBS and incubated with either Texas Red-conjugated rabbit anti-goat or FITC-conjugated donkey anti-mouse secondary antibodies. Two-dimensional images were collected and saved using a Nikon C1 scanning confocal imaging system.

### Co-immunoprecipitation

In order to verify the interactions among gamma-actin, non-muscle myosin IIB and FHL1 in L6GNR4 myoblastocytes *in vitro*, or E17 lower limbs *in vivo*, total protein extracts were prepared and immunoprecipitated as previously described [32]. Proteins were separated by SDS-PAGE (12% gel) and transferred to PVDF membranes for Western blot analyses. Membranes were blocked with 5% non-fat dried milk (NFDM) in TBST (20 mM Tris-HCl, pH 8.0, 150 mM NaCl, 0.05% [v/v] Tween-20) at room temperature for 2 h. Membranes were washed three times for 15 min each in TBST buffer, and then immunoreacted with primary antibody (anti-FHL1, 1∶2000; anti-actg1, 1∶2000;anti-myh10, 1∶300) in 5% NFDM/TBST at 4°C overnight. Next, the membrane were immediately washed three times for 15 min each in TBST buffer, and incubated with secondary antibody (goat anti-mouse, 1:10,000) in 5% NFDM/TBST at room temperature for 2 h. Finally, the membranes were washed three times for 15 min each in TBST buffer, and specific proteins were detected by reaction with the ECL Western blotting detection reagent (GE Healthcare).

### Immunofluorescence verification of co-localized proteins

The immunofluorescence procedure was performed as previously described except the sections were incubated overnight at 4°C in primary antibodies (anti-FHL1, sc-23176, 1∶100; anti-myh10, sc-376942, 1:100; or anti-gamma-actin, sc-65637, 1∶200).

## Results

### Identification of potential FHL1-interacting proteins

**Figure 1:**
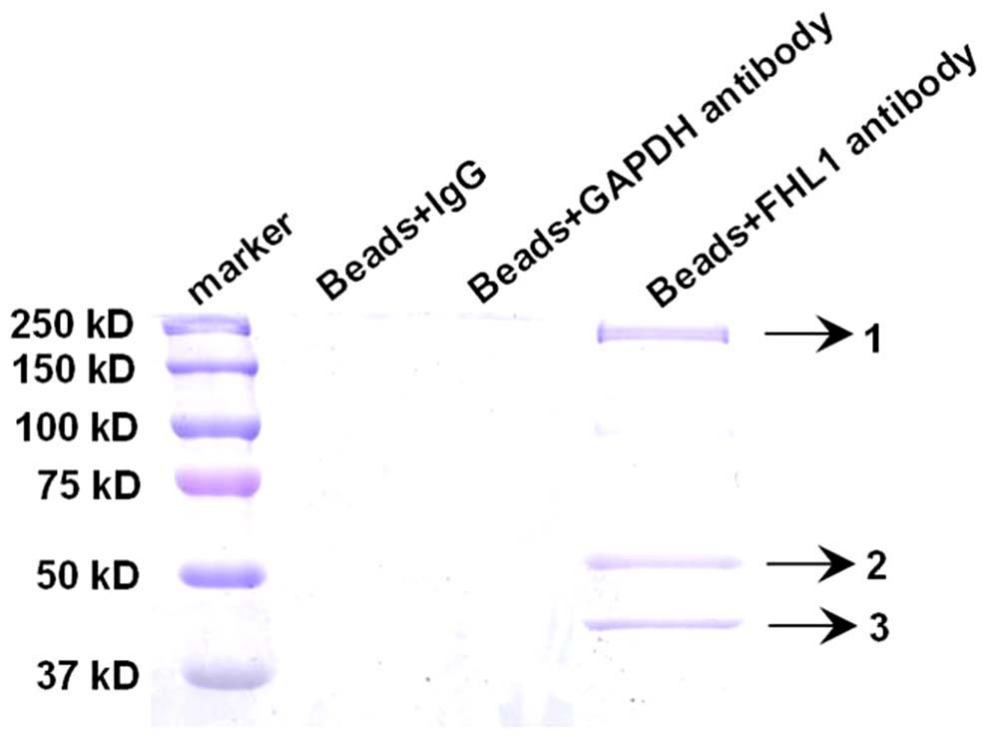
Immunoprecipitation followed by SDS-PAGE and CBB staining to detect protein binding to FHL1. Three bands were identified (denoted by arrows), which were not present in control samples.

**Figure 2:**
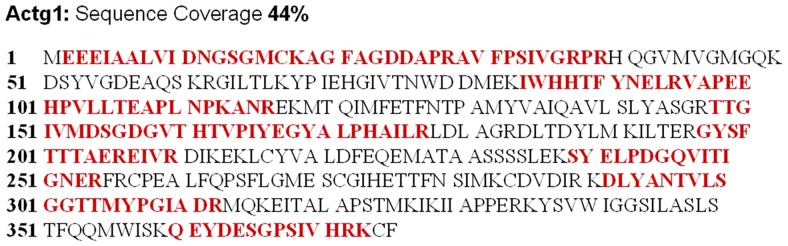
Mass spectrometric analysis of proteins binding to FHL1 identified gamma-actin by 19 peptide matches, which covered approximately 44% of the protein amino acid sequence. Red font indicates the matched amino acid sequences.

Proteins were isolated from L6GNR4 cells, immunoprecipitated, and analyzed by mass spectrometry to identify FHL1-interacting proteins. An FHL1 specific antibody identified three possible interacting protein bands with approximate molecular weights of 220 kDa, 50 kDa and 40 kDa (Fig. 1). These bands were digested by trypsin for subsequent MS analysis (see Materials and methods). The generated peptide spectra were searched against the rat IPI protein sequence database, and only those proteins, which were supported by at least two unique peptides per run were considered. When combined together, two different FHL1-interacting proteins were identified (Table 2). The peptide of interacting protein 3 covered 44% of the amino acid sequence identified as gamma-actin (*Actg1*) (Fig. 2) and the peptide of interacting protein 1 covered 19% of the amino acid sequence identified as non-muscle myosin IIB (*Myh10*) (see supplemental Fig. S1). MS analysis of the reported band 2 was identified as the tubulin alpha-1A chain. However, its MOWSE score was 41, which was lower than the accepted MOWSE score of 58. Thus, band 2 was not studied further.

**Table 2:** Proteins interacting with Fhl1 identified by MALDI-TOF MS.

Band	Gene	Protein name	Nominal mass	Matched peptide	Coverage	Score	Expect
1	*Myh10*	Non-muscle myosin IIB	228.936	40	19%	189	5e-015
*3*	*Actg1*	Gamma-actin	41.792	19	44%	246	1e-020

### Expression of FHL1 was associated with skeletal muscle differentiation

**Figure 3:**
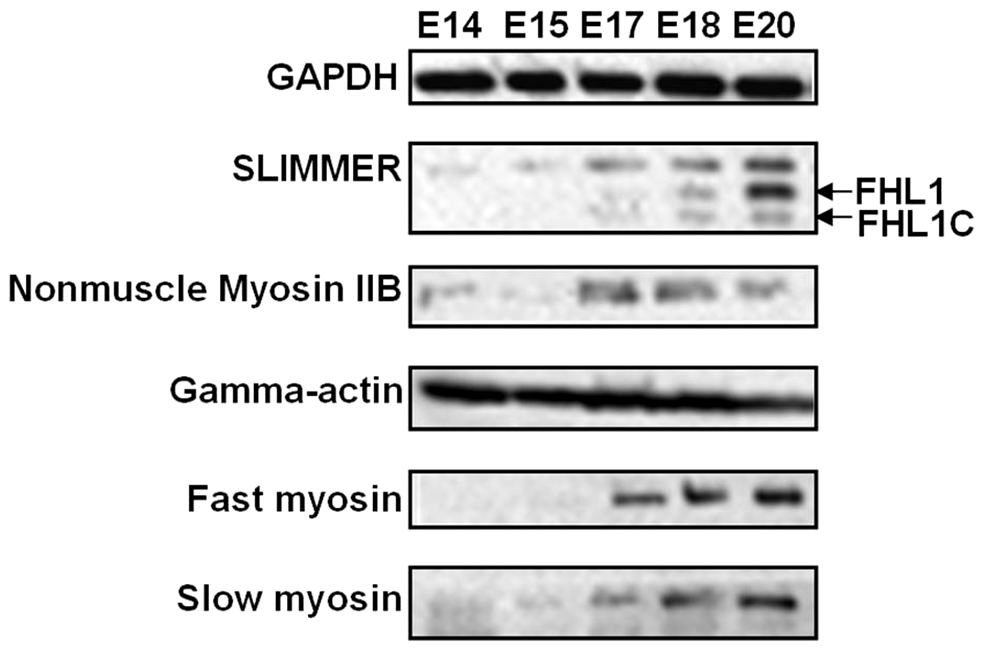
FHL1, gamma-actin, non-muscle myosin IIB expression in rat embryos. At E17, markers for skeletal muscle terminal differentiation (e.g. fast skeletal myosin and slow skeletal myosin) and expression of FHL1 were becoming evident, and the expression of the FHL1 interacting protein non-muscle myosin IIB achieved a peak at the same time. Slimmer, an isoform of FHL1, showed gradually increased expression with gestational duration.

**Figure 4:**
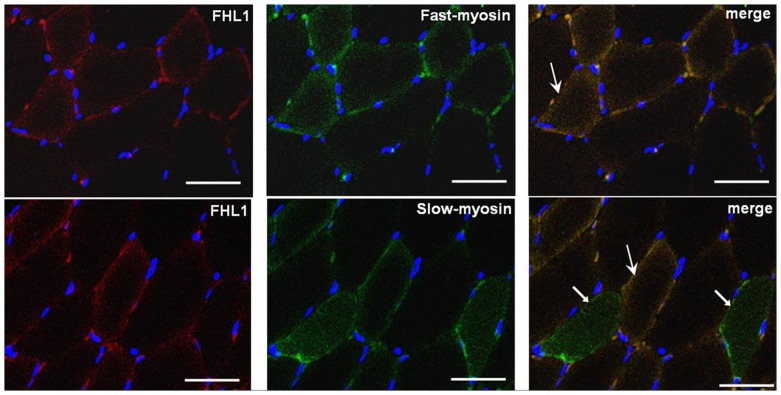
FHL1 expression in different muscle fibers of adult skeletal muscles. Large arrows indicate FHL1 and fast skeletal myosin positive fibers or FHL1 and slow skeletal myosin positive fibers. Small arrows indicate slow skeletal myosin positive fibers in which FHL1 expression was not found. Scale bar: 5 0 µm

In developing embryos, dynamic gene expression, and their interacting networks determine organ development and shape. Thus, we detected dynamic gene expression levels of FHL1, and determined the expression of the possible FHL1-interacting proteins gamma-actin and non-muscle myosin IIB in the lower limbs of E14, E15, E17, E18, and E20 rat embryos. Slimmer, an isoform of FHL1, showed gradually increased expression as a function of increases in gestational days. At E17, markers for skeletal muscle terminal differentiation (e.g. fast skeletal myosin and slow skeletal myosin) and expression of FHL1 were becoming evident, and the expression of the FHL1 interacting protein non-muscle myosin IIB achieved a peak at the same time (Fig. 3). In our unpublished data we found genes that control skeletal muscle development and differentiation (including *Pax3*, *Hgf*, *MyoD*, *Myogenin*) exhibited a peak in E17 lower limbs. In adult gastrocnemius muscles isolated from wild-type rats, we found that all of the fast skeletal myosin positive fibers expressed an FHL1 signal, and by contrast, partial slow skeletal myosin positive fibers showed expression of FHL1 (Fig. 4). As part of our current investigations of FHL1 function in skeletal muscle differentiation we found that slow skeletal myosin expression was downregulated in L6GNR4 cells (cultured in differentiation medium 48 h) after decreasing *Fhl1* expression through *Fhl1* specific siRNA transfection (data not shown). These observations indicated that variations in FHL1 expression were associated with skeletal muscle differentiation and that E17 is a critical time-point for skeletal muscle differentiation in the lower limbs of rat embryos.

### Expression of *Fhl1* and its potential binding partners in adult skeletal muscle

**Figure 5:**
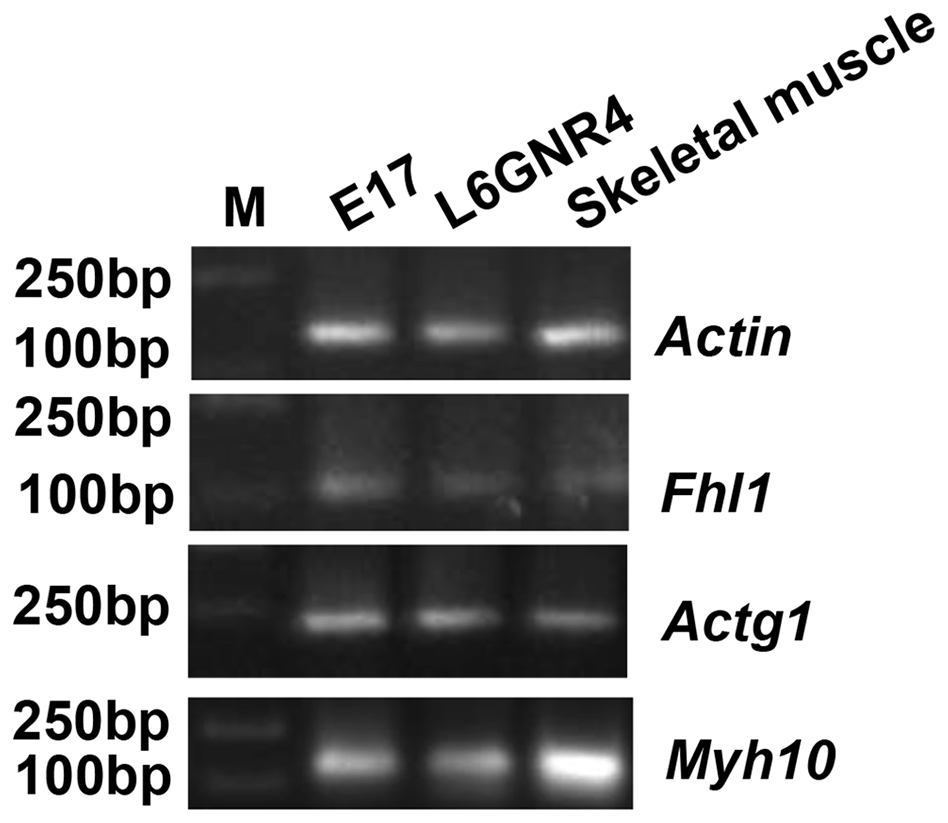
The electrophoretogram of RT-PCR products of *Fhl1*, *Actg1*, and *Myh10.* M represents the DL2000 DNA ladder. *Fhl1*, *Actg1* and *Myh10* expressed in adult skeletal muscle and positive controls (E17 lower limbs or L6GNR4 cells).

We found that *Fhl1*, *Actg1*, and *Myh10* RNA were expressed in adult skeletal muscle (Fig. 5). Our observations are consistent with those of Takeda et al. [31].

### Validation of interactions

**Figure 6:**
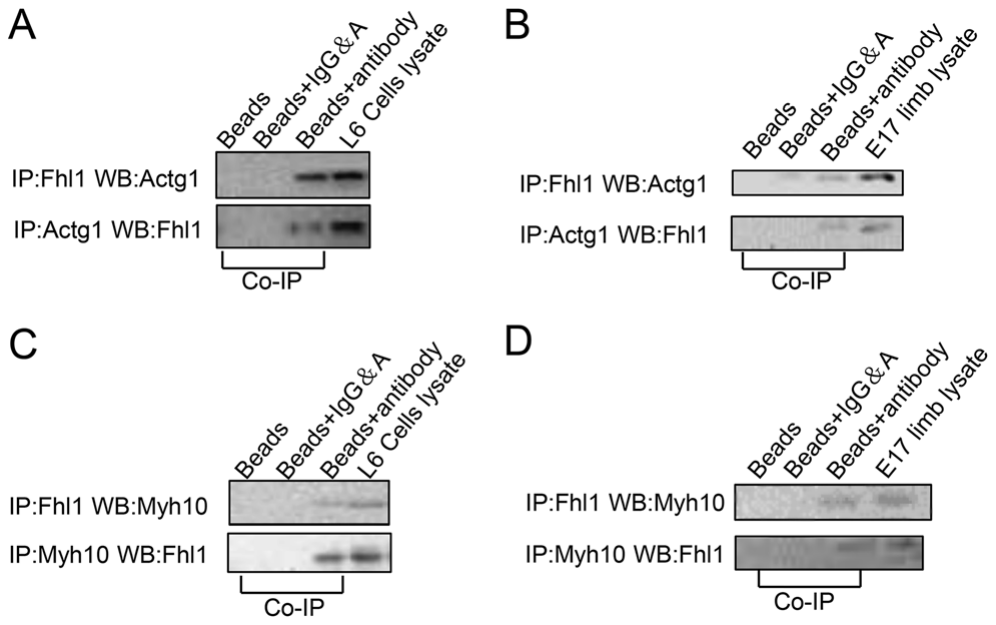
Validation of the potential interacting proteins with FHL1. L6GNR4 cell or E17 lower limb lysate was loaded as a positive control in immunoblots. **A:** L6GNR4 cells were immunoprecipitated using the anti-Fhl1 or anti-Actg1 antibody. Immunoblot detection verified that FHL1 co-immunoprecipitated with gamma-actin. **B:** Endogenous immunoprecipitation from wild-type E17 lower limbs using anti-FHL1 antibody co-immunoprecipitated with gamma-actin. **C:** L6GNR4 cells were immunoprecipitated using the anti-Fhl1 or anti-Myh10 antibody. Immunoblot detection verified that FHL1 co-immunoprecipitated with non-muscle myosin IIB. **D:** Endogenous immunoprecipitation from wild-type E17 lower limbs using anti-Fhl1 antibody co-immunoprecipitated with non-muscle myosin IIB.

Immunoprecipitation analyses were used to investigate the binding of FHL1 to either gamma-actin or non-muscle myosin IIB in cell lysates that were isolated from L6GNR4 cells. We found that FHL1 co-immunoprecipitated with gamma-actin and accomplished a reciprocal immunoprecipitation using an anti-Actg1 antibody to detect co-immunoprecipitation of gamma-actin with FHL1 (Fig. 6A). In skeletal muscle, FHL1 is involved in muscle differentiation, migration and growth. In this study we mainly focused on FHL1-interacting proteins involved in muscle differentiation and myotube formation, in which specific myosin heavy chains are expressed. At E17, markers for terminal skeletal muscle differentiation (e.g. fast skeletal myosin and slow skeletal myosin) and FHL1 were showing initial expression (Fig. 3). Our unpublished data showed that genes controling skeletal muscle development and differentiation (including *Pax3*, *Hgf*, *MyoD*, *Myogenin*) exhibited a peak in E17 lower limbs; thus we hypothesized that E17 is a critical time-point in skeletal muscle differentiation and myotube formation. Immunoprecipitation of wild-type E17 lower limb lysates confirmed the existence of the FHL1-gamma-actin complex at this stage *in vivo* (Fig. 6B). Additional co-immunoprecipitation analysis demonstrated that FHL1 also formed a complex with non-muscle myosin IIB (Fig. 6C, D).

### Verification of complex formation

**Figure 7:**
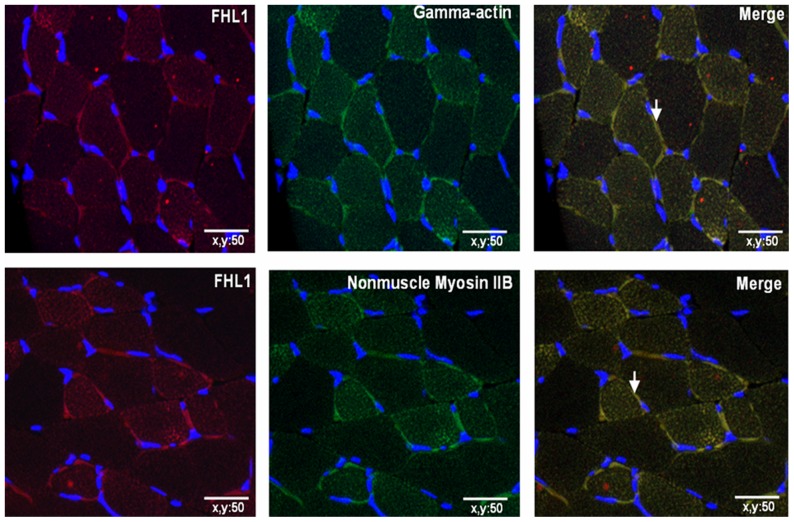
Immunofluorescence analysis showing co-localization of FHL1 expression with gamma-actin and non-muscle myosin IIB. Arrows indicate regions of colocalization, as indicated by the yellow colour.

Immunofluorescence analysis of wild-type adult rat gastrocnemius muscle tissues was performed to further verify complex formation between FHL1 either gamma-actin or non-muscle myosin IIB (Fig. 7). We showed that FHL1 co-localized with both gamma-actin, and non-muscle myosin IIB. This data provides indirect but further evidence of the existence of these complexes.

## Discussion

Four-and-a-half LIM domain protein 1 (FHL1) was identified as the founding member of the FHL family of proteins. FHL1 is characterized by the presence of four-and-a-half highly conserved LIM domains, which function as a modular protein binding interface to mediate protein-protein interactions. FHL1 displays several important functions ranging from developmental organization, muscle force transmission, and even a role in cell migration.

To date, more than 27 different protein interactions have been identified for full-length FHL1 and its splice variants, and these interactions have been mapped to a variety of functional classes [4], [14]. In skeletal muscle, FHL1 is involved in sarcomere assembly, muscle differentiation, growth, and in biomechanical stress responses [11]–[13]. Mice lacking *Fhl1* reversed the development of hypertrophic cardiomyopathy, which is induced by biomechanical stress. However, transgenic expression of *Fhl1* in mice promoted skeletal muscle hypertrophy [12], [13].

Muscle abnormality could be a key reason for CCF deformity, which manifests itself during fetal development [5]–[8], [10]–[29]. Thus studying FHL1 protein interactions would provide a greater insight into its functional role in skeletal muscle development and the pathogenesis of CCF or other myopathies induced by FHL1. In this study we used an immunoprecipitation approach to elucidate potential FHL1 binding partners. In this way we identified two FHL1-interacting proteins with a high degree of confidence by mass spectrometry. These interactions were studied further by co-immunoprecipitation and immunofluorescence, which demonstrated that FHL1 interacted as part of a complex with both gamma-actin (*Actg1*) and non-muscle myosin IIB (*Myh10*).

### Gamma-actin *(Actg1)*

Actins are a family of highly conserved cytoskeletal proteins that play fundamental roles in nearly all aspects of eukaryotic cell biology [33]. The ability of a cell to divide, migrate, endocytose, generate contractile force, and maintain shape is reliant upon functional actin-based structures. Higher eukaryotes express six distinct isoforms of actin, which are grouped according to their pattern of tissue expression: four ‘‘muscle’’ actins predominate in striated (α_sk_ and α_ca_) and smooth (α_sm_ and γ_sm_) muscle. By contrast, the two cytoplasmic ‘‘non-muscle’’ actins (β_cyto_ and γ_cyto_) are found in all cells [34]. While morphogenetic defects were not identified, *Actg1* (–/–) mice exhibited stunted growth during both embryonic and postnatal development, and they displayed a delay in cardiac outflow tract formation. However, *Actg1* (–/–) cells, exhibited growth impairment and reduced cell viability [35]. Many studies have shown that actin plays a key role in the regulation of apoptosis [36] and in *Actg1* (–/–) embryos, the loss of cyto-actin expression led to increased apoptosis. These observations provide a possible explanation for the reduced body size and delayed development in *Actg1* (–/–) embryos [35].

Cytoplasmic gamma-actin was found to be localized in the Z-discs of skeletal muscle, an observation which indicated its importance in skeletal muscle [33], [37]. In cultured myoblasts, β_cyto_ and γ_cyto_ are the predominant actin species present in these cells. However, upon myoblast fusion and differentiation, non-muscle actin mRNA expression is downregulated as a consequence of muscle isoform expression being switched on [38]. Transfection studies were designed to explore the role of disrupted expression of non-muscle actin. These studies showed abnormal cell shape in cultured myoblasts and myotubes. These observations suggest that muscle cytoskeletal architecture is highly influenced by the expression of gamma cyto-actin [39], [40].

To study the role of ACTG1 in skeletal muscle development, Sonnemann et al. conditionally ablated *Actg1* expression in mouse skeletal muscle. Although loss of gamma cyto-actin did not impede muscle development, muscle-specific gamma cyto-actin knockout (*Actg1*–*/*–) mice exhibited overt symptoms of skeletal myopathy including decreased mobility, limb weakness, and joint contractures. In addition, a progressive pattern of muscle cell necrosis, regeneration and significant force deficits were observed. These observations demonstrate a role for gamma cyto-actin in maintaining muscle cellular structure [37].

### Non-muscle myosin IIB *(Myh10)*

Non-muscle myosin and actin are thought to play important roles in cell motility, adhesion and cell shape [41]. The actin-myosin based cytoskeleton is a dynamic system essential for contraction, motility, and tissue reorganization [42]–[44]. Non-muscle myosin II is implicated in a variety of cellular processes, including cell migration, establishing cell polarity, cytokinesis, and cell-cell adhesion [45]. In mammals, three genes encode the non-muscle myosin II heavy chains, and these are termed NMHC IIA, NMHC IIB (*Myh10*), and NMHC IIC [46].

NMHC IIB is required for embryonic rat peripheral nerve growth cone mobility at the borders of laminin stripes in response to signals from laminin-activated integrin receptors. In the absence of NMHC IIB, neurite outgrowth continues across laminin borders [47]. Pharmacologic or genetic inhibition of *Myh10* altered protrusive motility of spines, destabilized their mushroom-head morphology, and impaired excitatory synaptic transmission [48]. Graded knockdown of NMII in cultured COS-7 cells revealed that the amount of NM II-limited ring constriction [49]. Takeda et al. studied the development of myocardial cells in *Myh10*-ablated mice. It was shown that homozygous null mice exhibited 70% fewer, but larger, myocytes than heterozygous and wild-type mice, with a marked increase in binucleation [50]. In cultured embryonic mouse cardiomyocytes, NMHC IIB knockdown led to decreased N-RAP levels, which demonstrated that NMHC IIB plays a key role in cardiomyocyte distribution and N-RAP function in myofibril assembly [51].

NMHC II-B expression in adult skeletal muscle is controversial. Murakami et al. found that NMHC II-B expression in striated muscles of fetal and neonatal mice decreased to levels that were below the limit of detection by 3 weeks of age [30]. In addition, Takeda et al. reported NMHC II-B expression at the Z-lines of adult human skeletal muscle cells based on immunofluorescence analysis [31], which is consistent with our detection of NMHC II-B expression in adult skeletal muscle sections. Studies on the function of NMHC II-B in skeletal muscle development have been uncommon. However, the interaction of non-muscle myosins 2A and 2B with actin have been shown to altered cell movement, shape and adhesion in cultured myoblasts. Furthermore, non-muscle myosin 2B knockdown markedly inhibited tail retraction, increased cell length, and interfered with redistribution of nuclei in myotubes [52].

## Conclusion

In summary, FHL1 is expressed predominantly in skeletal muscle. Multiple functions have been attributed to FHL1, including sarcomere assembly, cytoskeletal remodeling, biomechanical stress response, muscle hypertrophy, and transcriptional regulation. In this study we found that the Z-disc protein FHL1 interacted as part of a complex with the Z-disc proteins, gamma-actin (*Actg1*) and non-muscle myosin IIB (*Myh10*) [15]. Z-discs delineate the lateral borders of sarcomeres, and are the smallest functional units in striated muscle. Z-discs were initially regarded as important structures only for mechanical stability. However, recent reports have indicated that Z-discs serve as a nodal point for general signaling, mechano-sensation and mechano-transduction [53]. The discovery of the potential binding partners (gamma-actin and non-muscle myosin IIB) of FHL1 should further our understanding of its function in skeletal muscle development. Abnormal expression of FHL1 or its binding partners (gamma-actin and non-muscle myosin IIB) could influence skeletal muscle cell movement, shape and adhesion [12]–[15], [33]–[40], [41], [42]–[52]. We hypothesize that abnormal expression or mutations of FHL1-binding partners (gamma-actin or non-muscle myosin IIB) participate in the pathogenesis of some FHL1-induced myopathies. This could result in symptoms associated with some of the FHL1-induced myopathies, including decreased mobility, limb weakness, and joint contractures. However, the Z-disc complex containing FHL1, gamma-actin, non-muscle myosin IIB, its functional capabilities in skeletal muscle development and its mechanism in CCF (or other myopathies) induced by FHL1 have not been delineated, and require further investigation.

## Supporting Information

Figure S1 **Mass spectrometric analysis of proteins binding to FHL1**. Proteins that interacted with FHL1 were identified as non-muscle myosin IIB by 40 peptide matches, which covered approximately 19% of the protein amino acid sequence. Red font indicates representative matched amino acid sequences.
(TIF)
