# Supplementary figures and images for: Identification of an FHL1 Protein Complex Containing Gamma-Actin and Non-Muscle Myosin IIB by Analysis of Protein-Protein Interactions
Source: PLoS One. 2013 Nov 12;8(11):e79551. doi: 10.1371/journal.pone.0079551 (PMC3827166; doi:10.1371/journal.pone.0079551)

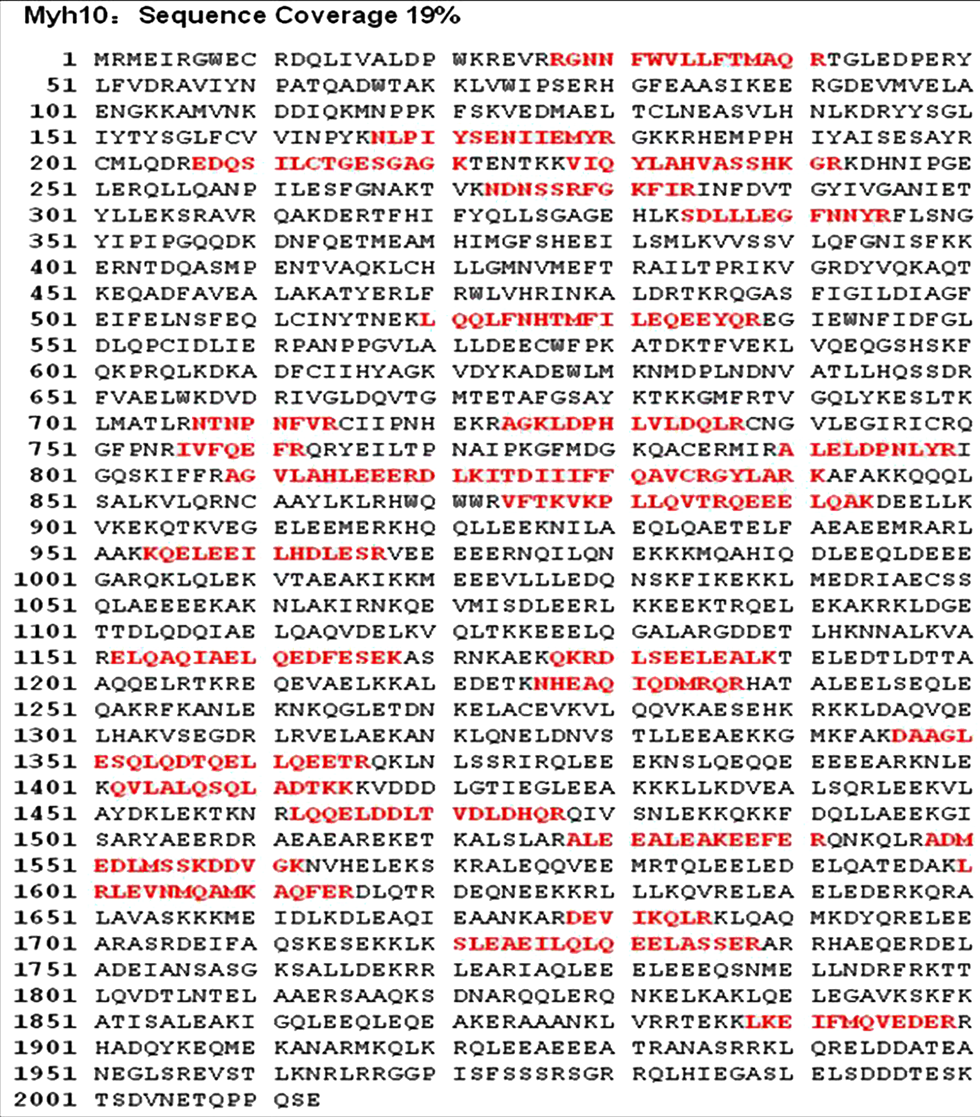

Supplement: Figure S1 — Mass spectrometric analysis of proteins binding to FHL1. Proteins that interacted with FHL1 were identified as non-muscle myosin IIB by 40 peptide matches, which covered approximately 19% of the protein amino acid sequence. Red font indicates representative matched amino acid sequences. (TIF) [file pone.0079551.s001.tif]
